# Securing Dynamic Service Function Chain Orchestration in EC-IoT Using Federated Learning

**DOI:** 10.3390/s22239041

**Published:** 2022-11-22

**Authors:** Shuyi Wang, Longxiang Yang

**Affiliations:** 1 College of Telecommunications and Information Engineering, Nanjing University of Posts and Telecommunications, Nanjing 210003, China; 2 Department of Information Engineering, Nanhang Jincheng College, Nanjing 211156, China

**Keywords:** IoT, edge computing (EC), federated learning, deep Q-network, SFC orchestration

## Abstract

Dynamic service orchestration is becoming more and more necessary as IoT and edge computing technologies continue to advance due to the flexibility and diversity of services. With the surge in the number of edge devices and the increase in data volume of IoT scenarios, there are higher requirements for the transmission security of privacy information from each edge device and the processing efficiency of SFC orchestration. This paper proposes a kind of dynamic SFC orchestration security algorithm applicable to EC-IoT scenarios based on the federated learning framework, combined with a block coordinated descent approach and the quadratic penalty algorithm to achieve communication efficiency and data privacy protection. A deep reinforcement learning algorithm is used to simultaneously adapt the SFC orchestration method in order to dynamically observe environmental changes and decrease end-to-end delay. The experimental results show that compared with the existing dynamic SFC orchestration algorithms, the proposed algorithm can achieve better convergence and latency performance under the condition of privacy protection; the overall latency is reduced by about 33%, and the overall convergence speed is improved by about 9%, which not only achieves the security of data privacy protection of edge computing nodes, but also meets the requirements of dynamic SFC orchestration.

## 1. Introduction

The era of the Internet of Things (IoT) is quickly approaching with the IoT’s rapid development in a variety of fields. However, with the development of business and the rapid increase in IoT devices, it has been gradually found that the method based on cloud computing cannot meet the actual needs of many scenarios. For example, huge amounts of data put great pressure on the network bandwidth, and the demand of networked devices for low latency and collaborative work increases, and these connected devices involve personal privacy and security. Edge computing (EC) arises at this historic moment; therefore, a large number of computational tasks near the source of the data processing greatly alleviates the pressure of network transmission, sharply reducing the time of data transmission in the network, increasing the speed of the users’ response times, and computational tasks will be able to be performed at the same time from the cloud after uploading to the edge, and the whole system of energy consumption will be reduced by 30–40%. Using multiple edge nodes to cooperate not only ensures an efficient solution to the problem, but also balances the problem of data privacy and the cost of data transmission in the network [1].

Due to the high requirement for the latency performance of emerging IoT services, suitable service orchestration is required to further decrease network end-to-end delay, enhance resource utilization, and lower deployment costs [2]. In a traditional network, the network function is highly coupled with the underlying physical hardware. When network equipment is deployed in the network, the data flow of the service must pass through the network equipment at a fixed location. Therefore, the deployment of additional network functions and services becomes more difficult and expensive as the network is scaled. When there is dynamic network traffic and continually shifting requirements, it is also challenging to provision them [3]. Network Function Virtualization (NFV) introduces a new set of management and orchestration functions in addition to the existing element management (EM) and operations support systems (OSS) functions [4]. NFV realizes the decoupling of network functions from dedicated hardware devices, and the Virtual Network Function (VNF) can be deployed to any location in the underlying network that satisfies resource constraints, which makes the updating and deployment of network services more economical and flexible. Combined with the centralized control and programmable characteristics of the Software Defined Network (SDN), network operators can easily implement VNF monitoring, management, and maintenance, greatly reducing the difficulty of network function deployment, and improving network performance and service [5]. In this way, network deployment and management can be implemented in an elastic, efficient, and flexible manner.

**Figure 1:**
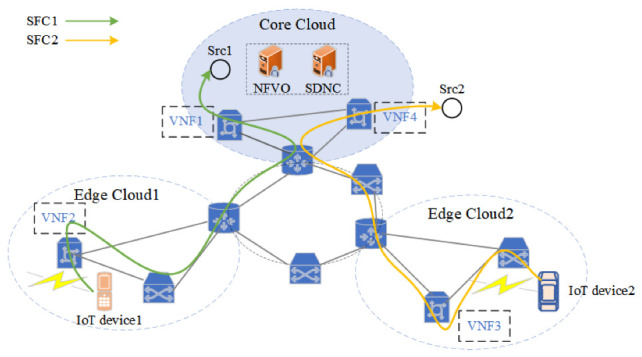
A running example of dynamic SFC orchestration in EC-IoT Network.

A Service Function Chain (SFC) is created by connecting a number of VNFs in a specific order via virtual links. Service data flows need to traverse the VNFs in order to complete end-to-end service delivery. SFC orchestration is the placement of the VNF and virtual connections in the SFC on the physical network as well as the distribution of physical network resources to them for end-to-end service delivery. Multiple VNFs can be deployed on a single server, allowing hardware resources to be shared in an efficient manner [6]. At the same time, due to the flexibility and diversity of IoT services, it is of great significance to study the dynamic orchestration technology of SFCs in the cloud-edge collaborative network application scenario. Figure 1 shows an example of dynamic SFC orchestration in an EC-IoT network [7]. In the figure, a core cloud and two edge clouds are deployed, and the IoT devices of the two edge clouds communicate with different sources through SFCs marked green and yellow, respectively. The NFV orchestrator (NFVO) and SDN controller (SDNC) are both deployed in the core cloud. The NFVO is mainly responsible for the VNF placement planning and VNF resource management in the SFC based on service requirements [8], and the SDNC is mainly responsible for SFC traffic engineering to realize SFC traffic communication. SDN technology dynamically directs traffic to pass via the preset, ordered VNFs; consequently, SFCs are produced depending on the constantly changing network service requirements [9]. Different network services that are provisioned in EC-IoT are represented by different colors in Figure 1. It is advisable to connect the edge clouds together when the number of IoT terminals rises in order to expose services to more nearby end users by effectively utilizing and sharing the capacity and load of edge clouds [10].

At present, most of the research on SFC orchestration focuses on solving the optimization problems of high reliability [11,12,13,14], low latency [7,10,15,16], cost-efficiency [17,18,19], energy-efficiency [20,21,22], scalability [23,24,25], and quality of service (QoS) [26] on the basis of satisfying the service functions; there is relatively less research on security [27,28,29,30], and the technology used in security-related research is mainly on blockchain. Since NFV offers software-enabled automated network function provisioning, it may potentially expose security flaws including automated network configuration exploits, orchestration exploits, malicious misconfigurations, and SDN controller exploits. At present, there are good solutions for some attacks against VNFs and platforms. For example, flexible VNF strategic deployment can be used to defend against a DDoS attack. A malicious insider can be defended by volume or swap encryption, VNF image signing, or strict operational practices. However, there are still some security challenges to be addressed. Managing trust between several manufacturers who produce NFV hardware and software is one of the issues. The difficulty lies in effectively managing the vendor trust chain and ensuring the reliability of the final VNF goods [3]. We must make sure that the service chain is established in a trustworthy manner when operating in a trustless environment because end-to-end SFCs may install VNFs in the territory of rival cloud providers. Additionally, an infrastructure with several tenants and domains makes it more likely for assaults to occur inside the cloud and makes it harder for service providers to be held accountable. Given that assaults on the host of VNFs have the potential to compromise thousands of users at once, the effects of potential attacks grow more severe [29].

Edge computing devices are more vulnerable to security threats due to tight physical connections with a large number of smart terminals and limited computing resources. Attackers typically target private data and rich digital assets on edge computing devices [1]. In current SFC orchestration schemes in edge computing scenarios, edge devices are mostly used to send local information to the core cloud for overall orchestration [7,31]. With the surge in the number of edge devices and the increase in data volume of IoT scenarios, there are higher requirements for the transmission security of privacy information from each edge device and processing efficiency of SFC orchestration. Blockchain is mainly applied to peer-to-peer transaction accounting and contracts to ensure that transaction records cannot be modified and that data storage will have certain redundancies [28,30], while federated learning is mainly applied to personalized user services, where data of each node can be invisible and there are no redundant data, which is more suitable for the application scenarios of EC-IoT [32]. Therefore, in this paper, the federated learning algorithm federated block coordinate descent scheme (FedBCD) [33,34] and deep reinforcement learning algorithm deep Q-learning network (DQN) [31] are combined to achieve the security and low latency performance of SFC orchestration.

In order to achieve the SFC orchestration effect of privacy security protection and communication efficiency on the basis of meeting the low latency requirements of the EC-IoT network, this paper adopts the quadratic penalty method to make the global model resist malicious attacks based on the federated learning framework [33,35]. The block coordinated descending method (BCD) is adopted to support the scenario of heterogeneous data and available computing power in different edge devices of users while meeting communication efficiency [33,34]. The edge cloud adopts the reinforcement learning method of DQN locally, takes low delay as the reward goal, and dynamically updates the SFC orchestration strategy according to the environmental change. According to the experimental results, it can be seen that compared to the SFC orchestration method, which only uses DQN [25] in the core cloud, the SFC orchestration method proposed in this paper can better provide privacy security protection ability. At the same time, the convergence can be achieved quickly on the basis of meeting the requirements of communication efficiency. The main contributions of this paper are described as follows.

- Federated learning framework combined with the BCD algorithm and the quadratic penalty algorithm is used to protect the private data during the dynamic SFC orchestration process in EC-IoT scenarios for the first time.
- In addition to realizing the privacy protection by the federated learning algorithm, the Deep Reinforcement Learning (DRL) algorithm DQN is used to dynamically generate the local SFC orchestration model of edge nodes by sensing the changes of requirements and environment in real time, at the same time, combining with the predictive network and target network mode, and setting up a replay buffer used to generate and develop reference samples for future learning in order to avoid the strong correlation between the training samples.
- By setting different network parameters to generate different random networks to simulate the EC-IoT environment, the convergence performance and delay performance of the proposed solution are verified from different dimensions including number of nodes, connection possibility, batch size, and number of SFC requests, and then compared with the mainstream dynamic SFC orchestration algorithm. Experimental results show that the proposed method can achieve good convergence performance and delay performance while preserving privacy.

In the second part, we will introduce the research situation of SFC orchestration in security. The third part introduces the proposed SFC orchestration approach in detail, the fourth part analyzes the experimental results, and finally summarizes the whole paper and puts forward the next research direction.

## 2. Related Work

In recent years, with the rapid development of SDN and NFV technology, more and more services are realized by way of service function chain orchestration. The VNF placement problem, SFC deployment problem, SFC resource allocation problem/SFC mapping or embedding problem [36], and SFC traffic engineering problem involved in the process of service function chain orchestration have become the hot spots of current research [37]. All these problems are proved to be NP-hard. Generally speaking, SFC orchestration can be divided into static orchestration in offline service scenarios and dynamic orchestration in online service scenarios. The current research is mainly based on subproblems in the process of SFC orchestration to achieve different objectives. It mainly includes the objectives of high availability and reliability, low latency, low cost or resource consumption [38], high energy utilization, scalability, and quality of service and security. This paper mainly studies the security and low latency in dynamic orchestration scenarios.

### 2.1. Enabling Technologies

#### 2.1.1. Network Function Virtualization (NFV)

**Figure 2:**
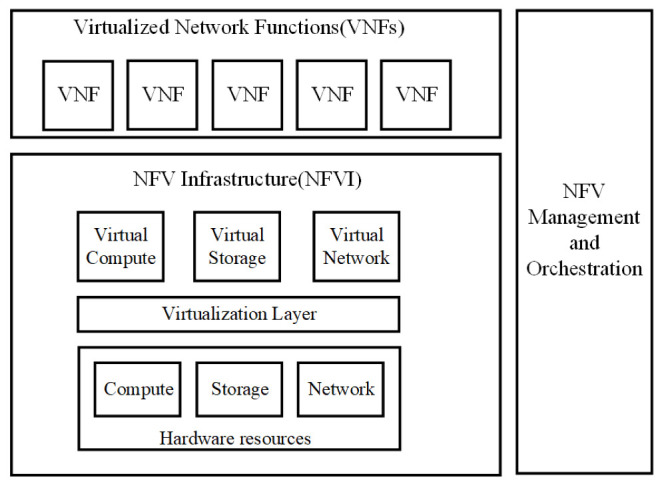
High-level NFV reference framework [4].

NFV is a potent, newly developed method with broad use. NFV envisions network functions being implemented as software-only entities that operate over NFVI. NFV is a step forward for the various stakeholders in the telecommunication network environment as compared to non-virtualized networks, where network functions are executed using a combination of vendor-specific software and hardware. In comparison to present practice, NFV brings a number of modifications on how network service provisioning is implemented. These differences can be categorized into three groups: decoupling software from hardware, flexible network function deployment, and dynamic operation. A larger degree of freedom is available to scale the real VNF performance in a more dynamic manner and with better granularity thanks to the decoupling of network function functionality into instantiable software components. Figure 2 shows the high-level NFV framework [4]. In NFV, three major working domains have been identified. First, VNF is defined as a network function’s software implementation that can operate over an NFVI network. The diversity of physical resources and their virtualization are covered by NFVI in part two. The execution of the VNFs is supported by NFVI. Third, NFV Management and Orchestration, which includes the lifecycle management of VNFs and the orchestration of physical or software resources that support infrastructure virtualization. It focuses on all management activities unique to virtualization that are required in the NFV architecture.

#### 2.1.2. Software-Defined Network (SDN)

SDN is a new network paradigm. In contrast to contemporary networks, where the IP layer merges both planes vertically into the network devices, its key characteristic is the separation of the network control plane from the data plane. The SDN Controller, a piece of software that represents the SDN control plane, is in charge of deciding how to handle the underlying network traffic in terms of network regulations and rules. Data forwarding according to a set of rules is the responsibility of the data plane, deployed as network devices. Through an Application Programming Interface (API) in the Northbound interface, the SDN controller permits the design and management of such rules. Through the protocols of the Southbound interface, it does have direct control over the components of the data plane. Such a division offers several undeniable benefits, including flexibility and simplification in the application of network policies, ease in network construction and development, and stimulation of creativity. Even though they serve diverse objectives, NFV and SDN represent complementary paradigms and technologies that can deliver a single integrated solution. In order to achieve this, SDN can automatically and flexibly provide connectivity between VNFs and the streamlining network administration. In this situation, SDN Controllers and Management Applications can both operate as VNFs in a scalable environment and obtain access to critical capabilities such as availability, reliability, and flexibility [39].

#### 2.1.3. Service Function Chain (SFC)

**Figure 3:**
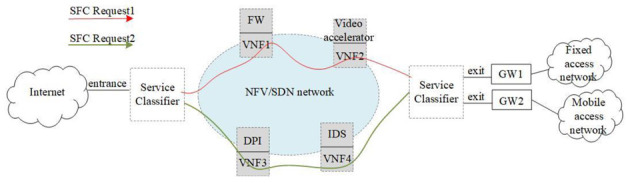
Logical diagram of SFC.

An SFC is a network technology that provides and manages special application services flexibly. It can classify the flow according to the service demand and network availability, and provide customized network services for users by running appropriate policies on the path of the flow. An SFC is composed of a series of VNFs arranged in a specific order. It requires network flows to be processed by VNF in order to achieve users’ specific network service needs [40]. The European Telecommunications Standards Institute (ETSI) defined a virtual network function forwarding graph (VNFFG) to describe the SFC service, and proposed the network functions virtualization management and orchestration (NFV-MANO) architectural framework to manage the NFVI and orchestrate the allocation of resources needed by the network services and VNFs [8]. The Internet Engineering Task Force (IETF) has completed a series of RFCs and drafts for SFC, covering the SFC architecture [41], SFC problem [42], and specific use cases such as data centers [43]. Figure 3 shows the logical diagram of an SFC in an NFV/SDN network. After the user submits an SFC request (SFCR) to the operator, the operator’s orchestration layer classifies the requirements to generate specific SFCs and maps them to different SFC paths according to the required VNF sequence. Figure 3 contains two SFCs in total. The order of the SFCs in the red line is the entrance node, the firewall, the video accelerator, and the exit node. The order of SFCs in the green line is the entrance node, deep packet detection, intrusion detection system, and exit node. If the user submits a red line SFCR service request to the operator, the network operator will configure the SFC path of the service for the user, so that the user’s network flow passes through the firewall and the video accelerator in order, and finally is transmitted from the exit node to the user terminal. The SFC is an important part of end-to-end network services, and it is also a supporting technology for implementing various network function combination services in network slicing. Research on the resource adaptation mechanism of dynamic SFCs is very important for network operators. Flexible function combination and efficient resource allocation are the keys to meet the diversified service demands in the future.

### 2.2. Security Research

Existing security schemes are mainly implemented by blockchain technology, a few researchers use SSL/TLS encryption technology to achieve the security effect [44]. Here, we focus on security solutions implemented using blockchain.

Igor D. Alvarenga et al. [27] mainly focus on the security attack problem of the core part of the network and propose a security architecture of managing, configuring, and migrating VNFs based on blockchain, which can ensure the security of the migration and updates of the core configuration of the network. Based on the research in the literature [27], Gabriel Antonio F.Rebello et al. [29] suggest using blockchain and transaction architecture to give traceability in an NFV context with several tenants and domains. The Practical Byzantine Fault Tolerance (PBFT) consensus protocol is simplified and used in the Open Platform for Network Function Virtualization (OPNFV) to build the BSec-NFVO approach. It can result in stable performance as the number of consensus participants rises and reduces the overheads for the cloud coordinator. These two methods mainly focus on the security of the core cloud but do not consider the communication efficiency between the edge cloud and the core cloud, and the protection of data privacy of the edge cloud.

Shaoyong Guo et al. [28] introduce a consortium blockchain and DRL algorithm Asynchronous Advantage Actor-Critic (A3C) to build a trusted, automatically tunable SFC orchestration architecture. To enable the sharing of trustworthy resources, this design incorporates the consortium blockchain into the distributed SFC orchestration mechanism. In the literature, Shaoyong Guo et al. [30] build a model for heterogeneous IoT network resource management based on the consortium blockchain and suggest a workable Byzantine fault-tolerant consensus method based on reputation value, which lowers consensus costs and boosts efficiency. To reduce orchestration expenses, a service function chain orchestration method based on A3C is created. The DRL method is introduced here for cost optimization and resource management of the SFC orchestration; the main consideration is the centralized management of the overall level of safety, but as the data privacy protection does not take into account the edge computing nodes, related data information at the edge of the device are needed to transfer to the core of the global cloud orchestration layer for orchestration.

It has been demonstrated that using blockchain technology to secure the integrity of data exchanges between entities is successful. The data will remain unchangeable and accessible to larger applications after the transaction has been added to a blockchain block for reasons pertaining to their business logic [45]. However, some privacy concerns may surface depending on how blockchain is used, making it feasible to track the transactions of a specific entity. The blockchain could be used by malicious players on an equal footing, endangering the accurate identification of IoT devices. Anonymization is typically utilized in blockchain technology applications to offer privacy protection, but it also raises some traceability concerns that might reveal the true identities of the blockchain participants in the transaction. The significant inter-operational and computational expenses should also be emphasized as undesirable elements. Given the significant heterogeneity of IoT devices participating in blockchains, there is a greater possibility that the information at stake will not be taken seriously [46].

Google created the idea of federated learning for on-device learning and data privacy protection. The method allows each IoT device to train its model using data that are obtained locally. It is not necessary for IoT devices to transmit local data to a centralized cloud. The updated local training model only needs to be collected by the centralized cloud from certain users. The privacy and security are guaranteed because the devices taking part in federated learning do not have to exchange their data samples. The fact is that federated learning preserves data privacy and uses less power and latency than standard machine learning methods [32]. Considering the characteristics of EC-IoT scenarios and the computational cost, this paper adopts the federated learning method to achieve privacy protection.

In this paper, the research focus is different from the existing SFC orchestration security scheme; we use the federated block coordinated descent algorithm to protect the privacy of edge nodes, as it has been proved that the algorithm can protect the privacy of a heterogeneous network environment while ensuring communication efficiency [33,34]. Although the author also used the federated learning framework in the literature [25], he focused on the scalability of SFC orchestration, and did not optimize the security aspect. In addition, the federated learning algorithm adopted in it did not pay much attention to communication efficiency, but mainly focused on cost minimization.

### 2.3. Dynamic SFC Orchestration Research in EC

In the beginning, the traditional optimization algorithm was used to optimize the SFC orchestration problem. In the literature [16], a dynamic minimum response time considering the same level is proposed by Gang Sun et al. to efficiently map the workflow-like service request in EC. In the joint optimization framework, online optimization techniques and approximate optimization methods are combined by Zhi Zhou et al. to maximize holistic cost efficiency [18]. Song Yang [10] proposes a delay-aware efficient randomized rounding approximation algorithm to solve the VNF placement and routing problem. Defang Li [19] models the VNF placement problem in EC as an ILP model, and proposes an efficient polynomial time heuristic to solve it. Although traditional optimization algorithms can achieve some optimization objectives, they cannot well meet the dynamic and flexible service orchestration scenarios in the current IoT environment [47].

In recent years, with the continuous development of machine learning technology, a large number of researchers began to apply machine learning to the dynamic orchestration of SFC because of its intelligent learning and prediction ability.

Tejas Subramanya et al. [48] create a classifier and a regressor that are neural-network-based MLP models to identify and exploit hidden patterns in network traffic load instances to predict user plane function (UPF) scaling decisions ahead of time. Yicen Liu et al. [49] propose a quantum machine learning (QML)-based algorithm to handle complex and dynamic SFC orchestration in mobile edge cloud networks. The authors use a Quantum Evolution with Feedback (QEF) algorithm to minimize the end-to-end delay during the dynamic SFC orchestration process in edge computing scenarios. The original machine learning algorithms cannot well adapt to the dynamic changes of service requirements in EC-IoT scenarios. Therefore, reinforcement learning algorithms, which can dynamically adjust orchestration strategies based on environmental changes, are applied to SFC orchestration scenarios. Yicen Liu et al. [7] use a DRL-based algorithm DDPG to solve the dynamic SFC orchestration problem. However, it still transfers the data of edge nodes to the core cloud node for training, without considering the privacy protection of edge nodes.

In this paper, the DRL method based on DQN is adopted to achieve dynamic SFC orchestration by combining the target network and replay buffer mechanism to conduct model training on edge nodes locally, and then the block coordinated descent algorithm based on federated learning is adopted to achieve privacy protection of edge nodes while ensuring communication efficiency. The privacy security of edge nodes is considered. It can also adapt to the scenario of dynamic orchestration of IoT services.

## 3. The Proposed Approach

### 3.1. Problem Statement

SFC orchestration refers to the process of analyzing and modeling according to the user’s service requirements, deploying service function (SF) instances, resource allocation management, service function chaining, and finally achieving single or multiple service goals. After the service demand is dynamically adjusted, the SFC also needs to be adjusted simultaneously. The SFC orchestration (SFCO) problem studied in this paper is to find the solution to achieve the optimal goals.

**Figure 4:**
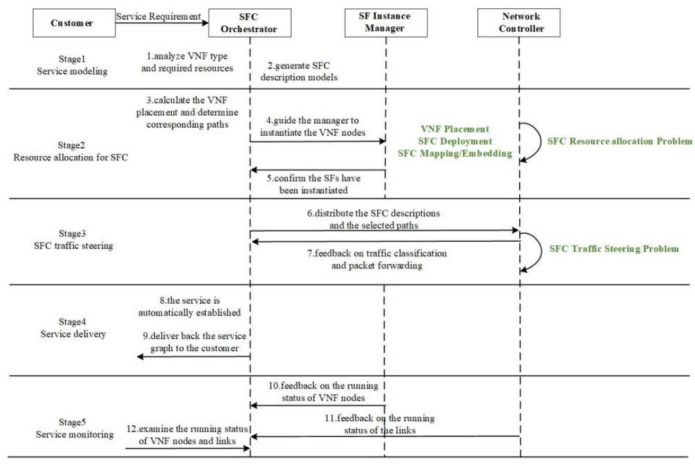
The stages of SFC orchestration process.

In the literature [50], the workflow process of SFC Orchestration in an NFV/SDN network is presented, and the sub-problems of SFC orchestration during the workflow process are described in the literature [37]. The name of each sub-problem has different words in academic circles, but the process correspondence is consistent. Therefore, this paper introduces the corresponding sub-problems according to each stage of the SFC orchestration process, as shown in Figure 4. The SFC orchestration process has five stages: Service modeling, Resource allocation for SFC [51], SFC traffic steering, Service delivery, and Service monitoring. In the case of the unified orchestration of the core cloud, the SFC orchestration is only deployed in the core cloud. In this scheme, in addition to the unified SFC orchestration in the core cloud, the agent of the SFC orchestration is also deployed in the edge cloud.

#### 3.1.1. Network Model

The underlying physical network is where the SFC is deployed. Typically, a physical network consists of a number of servers linked together via switches and related physical network lines. Both the server and the connection have certain computing and bandwidth resources. Physical links are abstracted as links in the topology and servers are abstracted as nodes in the modeling process.

The physical network can be modeled as $G^{P}=\left(N^{P},L^{P},Cap^{N}\right)$, where $N^{P}=\left\{n_{1}\right.,n_{2},\cdots ,\;\left.n_{\left|NP\right|}\right\}$ is the set of network nodes, $L^{P}=\left\{l_{1}\right.,l_{2},\cdots ,\left.l_{\left|LP\right|}\right\}$ is the set of network links, and $Cap^{N}=\left\{cap_{1}\right.,cap_{2},\cdots ,\left.cap_{\left|CAPN\right|}\right\}$ is the node resource capacity set of different nodes. The number of network nodes is denoted by $\left|NP\right|$, the number of physical links is denoted by $\left|LP\right|$, and $\left|CAPN\right|$ refers to the number of node resource capacity; it is less than or equal to $\;\left|NP\right|$. Typically, a server with specific computational capacity, such as $PR_{n_{m}}^{mem}$ and $PR_{n_{m}}^{cpu}$, is referred to as a network node $n_{m}$. The node’s remaining computational capacity is represented by $ResPR_{n_{m}}^{mem}$ and $ResPR_{n_{m}}^{cpu}$, respectively, $PR_{l_{n}}^{bw}$ denotes all bandwidth resources for a physical link $l_{n}$, while $ResPR_{l_{n}}^{bw}\;\mathrm{denotes}$ the remaining bandwidth resources. $p_{m_{i}m_{j}}$, a subset of $L^{P}$ that contains all the links on a path from node $n_{m_{i}}$ to node $n_{m_{j}}$, is also used to signify the path from node $n_{m_{i}}$ to node $n_{m_{j}}$. $n_{l_{n}^{1}}$ and $n_{l_{n}^{2}}$ refer to the nodes that are correspondingly connected at the link’s ($l_{n})$ two ends. Thus, the link’s $\left(l_{n}\right)$ transmission delay is indicated by $PND_{l_{n}}$ or $PND_{l_{n}^{1}l_{n}^{2}}$. The total delay of all physical links on this path, represented as $PND_{m_{i}m_{j}},\;\mathrm{is}$ the end-to-end delay from $n_{m_{i}}$ to $n_{m_{j}}$.

#### 3.1.2. SFC Model

An SFC request consisted of a set of SFs and links based on the user’s actual requirements. In the NFV scenario, the SF corresponds to VNF. Both the virtual network link and the VNF make specific bandwidth and computational resource requests. The requested resources must be larger than the remaining resources of the deployed node or link. The VNF has stringent order requirements as well. From terminals to users, traffic must move in a specific order. One may think of the SFC as a single linked list.

An SFC request $SFCR_{i}\in SFCR$ can be generally presented using a 7-tuple $\left\{Src_{i}\right.,\;Dst_{i},\;VNF_{i},\;R_{i}^{bw},\;R_{i}^{mem},\;R_{i}^{cpu},\left.P_{i}^{maxd}\right\}$, where $Src_{i}$ and $Dst_{i}$ refer to the ingress node and egress node, respectively. The set of VNFs requested by SFC request $SFCR_{i}$ is denoted by $VNF_{i}=\left\{VNF_{i}^{1},\;VNF_{i}^{2},\;\cdots ,\;VNF_{i}^{j}\right\},\;j=\left|VNF_{i}\right|$, where $VNF_{i}^{1}$, $VNF_{i}^{2}$, ⋯,$\;VNF_{i}^{j}$ represent the $1^{st},2^{nd},\cdots ,j^{th}$ VNF requests in $VNF_{i}$, respectively. The demands of bandwidth, memory, and CPU on links, nodes and VNF instances are denoted by $R_{i}^{bw}$,$\;R_{i}^{mem}$, and $R_{i}^{cpu}$, respectively. $P_{i}^{maxd}$ refers to the maximum tolerated delay of SFC request $SFCR_{i}$. The parameters here are general requirements indicators, which can be expanded according to actual requirements.

The set of SFCs is denoted by $\;SFC_{List}=\left\{G_{1}^{SFC}\right.,G_{2}^{SFC},\cdots ,\left.G_{\left|SFC_{List}\right|}^{SFC}\right\}$, where $\left|SFC_{List}\right|$ represents the number of SFCs. An SFC is modeled as a directed weight graph $G_{k}^{SFC}$=$\left(N_{k}^{S},VL_{k}^{S}\right)$, where $N_{k}^{S}=\left\{vnf_{k}^{1}\right.,vnf_{k}^{2},\cdots ,\left.vnf_{k}^{\left|NS\right|}\right\}$ refers to the set of VNFs in the SFC, and $VL_{k}^{S}=\left\{vl_{k}^{1},\right.\;vl_{k}^{2},\;\cdots ,\;vl_{k}^{\left|VLS\right|}\}$ refers to the set of virtual network links. $\left|NS\right|$ and $\left|VLS\right|$ reflect the number of VNFs and links in the SFC, respectively. A particular amount of computer resources, such as $R_{vnf_{k}^{i}}^{mem}$ and $R_{vnf_{k}^{i}}^{cpu}$, are needed to deploy a VNF, and a similar amount of bandwidth resources, such as $R_{vl_{k}^{i}}^{bw}$, are needed to build a virtual network link $vl_{k}^{i}$. The total resource usage of the VNF with type t is denoted by $R_{vnf_{k}^{i}}^{t}.\;$ Each SFC has known source node and destination node, which are represented by $SFC_{k}^{src}$ and $\;SFC_{k}^{dst},\;\mathrm{respectively}$. The source node and the destination node stand for the terminal and the user, respectively. In addition, the predefined order, $VNFchain_{k}=\left\{vnf_{k}^{1}\right.\to vnf_{k}^{2}\to \cdots \to \left.vnf_{k}^{\left|NS\right|}\right\},$ shall be followed when traversing VNFs.

#### 3.1.3. Objective and Constraints

The objective of this paper is to achieve the optimization effect with the lowest latency. Here, we use the transmission delay to measure. The constraints are that the resource capacity of SFC deployment should be less than the total resource capacity of physical nodes, the bandwidth of virtual links should be less than the total bandwidth of physical links as well. The formula for the goal and constraints are as follows.

(1) $$\min:\;TDelay_{t}=\sum_{j=1}^{m}\frac{pktsize}{ResPR_{l_{n}}^{bw}}\;t\in T$$

Constraints:

(2) $$\sum_{j=1}^{m}f_{p}^{j}\left(t\right)R_{vnf_{k}^{j,p}}^{mem}\leq PR_{p}^{mem},\forall p\in N^{P},t\in T$$

(3) $$\sum_{j=1}^{m}f_{p}^{j}\left(t\right)R_{vnf_{k}^{j,p}}^{cpu}\leq PR_{p}^{cpu},\forall p\in N^{P},t\in T$$

(4) $$\sum_{i=1}^{n}f_{pq}^{i}\left(t\right)R_{vl_{k}^{i,p,q}}^{bw}\leq PR_{pq}^{bw},\forall pq\in L^{P},t\in T$$

The VNF instance number of SFC k is denoted by m, the data size of service packets is denoted by pktsize, and the remained bandwidth of physical links is denoted by $ResPR_{l_{n}}^{bw}$. The total transmission delay of a SFC at time t is denoted by $TDelay_{t}$. The constraints illustrate that the required resources of VNF instance j and link pq are no more than the maximum resource of node p and the bandwidth capacity of link pq, respectively.

### 3.2. Overall Framework Design

**Figure 5:**
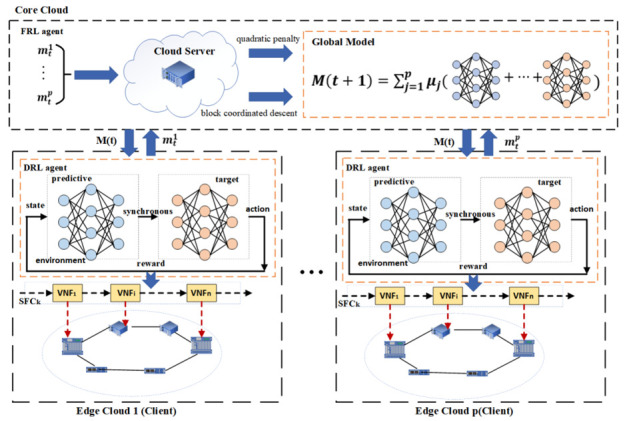
The overall framework of proposed approach.

The overall framework of the proposed method is shown in Figure 5. In order to protect the privacy of edge cloud node data, the federated learning framework is adopted to deploy the DRL agent in the edge cloud and the FRL agent in the core cloud, respectively. The core cloud belongs to the server side, and each edge cloud belongs to the client side.

Firstly, the FRL agent on the cloud server uniformly sends the initial network model to each edge cloud client, and then the DRL agent on the edge cloud client trains the received neural network model by dynamically sensing the environmental state changes, and obtains the actions of SFC orchestration, with the goal of minimizing end-to-end delay as the reward. The stability of the trained neural network model is improved by the mechanism of the regular synchronization of parameters between the prediction network and the target network. At the same time, the experience replay buffer is used to reduce the data correlation, which makes the samples reusable and improves the learning efficiency.

**Figure 6:**
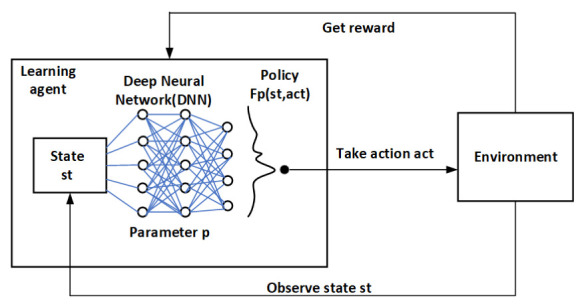
The Deep Q-learning Network model.

The DRL algorithm used in the edge cloud is DQN. DRL combines the perception ability of deep learning with the decision-making ability of reinforcement learning, which is an end-to-end perception and control system with strong generality. The action value in each state is recorded using a Q-table by the Q-learning algorithm. The storage space will be huge whenever a large state space or action space exists. The algorithm cannot be utilized if the state space or action space is continuous. Therefore, discrete low-dimensional state space and action space issues are the only ones that the Q-learning technique can be utilized to solve. The key component of the DQN method is the substitution of an artificial neural network for the action value function of the Q-Table. Each action will be the output value once the network has received the state information. Therefore, issues involving discrete action space and continuous state space can be solved using the DQN algorithm. The DQN model is shown in Figure 6. The DQN algorithm is an off-policy algorithm. Convergence cannot be guaranteed in the presence of alternatives, function approximation, and self-interest at the same time, and issues such as unstable training or challenging training are simple to develop. To address these issues, researchers primarily improved on the following two areas: target network and experience replay [52].

The goal of the target network is to replicate the original neural network’s exact structure. The initial network and the freshly built network are equivalent to the predictive network and the target network, respectively. The target network is employed to carry out evaluation values that are both self-helpful and gratifying as learning objectives during the learning process. The target network’s weight is not updated during the update process, only the predictive network’s weight is. The weight of the predictive network will be copied to the target network and used throughout the update process of the following batch once the target network has been updated for a predetermined number of times. The addition of the target network can increase the stability of learning because the estimation of rewards is relatively fixed across time and the target network is unchanging. Experience replay is to store experiences (current state $st_{t}$, action $act_{t}$, immediate reward $rew_{t+1}$, next state $\;st_{t+1}$, and turn state *done*) in the experience pool and sample according to certain rules. It is a technique to stabilize the empirical probability distribution, which can improve the stability of training.

After several rounds of local training for each edge cloud node, the locally trained parameter model is then transmitted to the cloud server of the core cloud for federated reinforcement learning. The new global model obtained by the cloud server is sent to each edge cloud node for a local training update until the iterative training reaches convergence and stability. In the FRL agent, the block coordinated descent algorithm and quadratic penalty method are also used to ensure the communication efficiency and enhance the security of the training model data.

(5) $$\underset{\begin{array}{c} \left\{em_{i}\right\}i\in \rho_{n,}n\in C\\ \left\{cm_{n}\right\}n\in C \end{array}}{\min}\sum_{n\in C}\sum_{i\in \rho_{n}}\left(f_{i}\left(em_{i}\right)+\frac{\delta_{i}}{2}\mathrm{||}em_{i}-cm_{n}\mathrm{||}{}^{2}\right)$$

(6) $$f_{i}\left(em_{i}\right):=\frac{1}{\left|V_{i}\right|}\sum_{\theta \in V_{i}}h\left(em_{i};v_{\theta }\right)$$

In Formula (5), *C* is the set of cloud servers,$\;\rho_{n}$ is the set of edge devices connected to the *n*th cloud server, $em_{i}$ is edge-device model, $cm_{n}$ is cloud-server models, and $\delta_{i}>0$ is the penalty parameter. In the sequel, $cm_{n}$ will be referred to as the global model, and $em_{i}$, the personalized model. In Formula (6), *h* is the training loss function, $V_{i}$ is the index set of training data on the *i*th edge device, $\left|V_{i}\right|$ is the number of elements in the set $V_{i}$, and $v_{\theta }$ is one of such samples.

Here, we take the resources of each node of the network as the current state space $S\left(t\right)$, and the resources include the CPU, memory resources of the node, and bandwidth resources on the link, forming a triple. The action obtained from the network model trained according to the current state data can be understood as the network node on which VNF is deployed. The action space is a set of network node numbers, and the reward is the reciprocal of the corresponding delay multiplied by the coefficient of reward and punishment β.

(7) $$S\left(t\right)=\left\{\left\{PR_{n_{1}}^{mem},\ldots ,PR_{n_{m}}^{mem}\right\},\left\{PR_{n_{1}}^{cpu},\ldots ,PR_{n_{m}}^{cpu}\right\},\left\{PR_{l_{1}}^{bw},\ldots ,PR_{l_{n}}^{bw}\right\}\right\}$$

(8) $$action\left(t\right)\;\in \;N^{P}$$

(9) $$reward\left(t\right)=1/TDelay_{t}\times \beta$$

## 4. Experimental Results and Analysis

Next, we will introduce the experimental process and analyze the experimental results.

### 4.1. Experiment Environment

Due to the random distribution of the underlying topology of the IoT infrastructure, it is difficult to manage during the SFC orchestration process. In order to verify the availability and generality of our proposed algorithm, considering the substrate networks of IoT can be complex and varied, we choose random networks to conduct the experiments. The random distribution of nodes in these networks can better reflect the irregularity of the IoT topology and the performance of our algorithm can be better evaluated.

**Figure 7:**
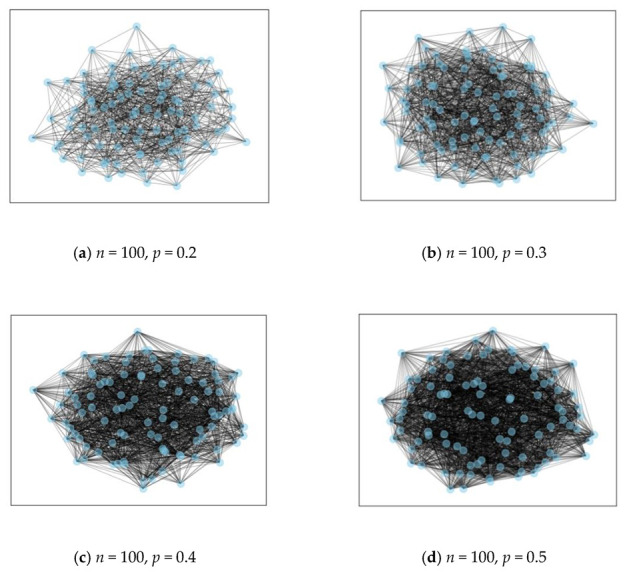
Random network examples.

The distributions of random networks in 100 nodes with different parameters are shown in Figure 7, respectively. The bandwidth capacity is assumed in range from 30 to 50 Mbps. Many kinds of physical networks can be represented by random network, such as ISP networks. Given a fixed number of nodes, and a fixed probability that there is an edge between the nodes, such a method generates a network relation, which is the mainstream G (N, p) random network. The connectivity probability relies on the distance between a pair of nodes. Figure 7 present the examples of random network with 100 nodes, where the connectivity probability range is from 0.2 to 0.5.

In order to be closer to the actual IoT resource usage scenario and improve resource usage efficiency, we consider the dynamic change in network load and resource usage over time. The IoT node generally refers to the sensor; in some scenarios, there can also be an RFID read/write device. Most of the nodes in EC-IoT are LPWAN nodes, so the resource requirements of experimental nodes are set according to the requirements of LPWAN IoT nodes. The parameter settings in our experiments are show in Table 1. The computing resource of the underlying node is set randomly, ranging from 1 to 8 GHz. The memory resource is set in the range from 4 to 64 GB, and the bandwidth capacity is set in the range from 30 to 50 Mbps. The resource demands of VNF are generated randomly considering the remaining available resources of nodes.

In our simulations, NetworkX 2.8 (Centrum Wiskunde & Informatica, The Netherlands) is used to simulate the underlying network topologies of IoT, and Pytorch (Linux Foundation, San Francisco, CA, USA) is used to implement FRL and DRL network. The software we used included Pycharm Community Edition 2021 (JetBrains, Prague, Czech Republic) and Anaconda3 with Python 3.7 (Centrum Wiskunde & Informatica, The Netherlands) on Windows 10.

### 4.2. Results and Discussions

After constructing the network topology environment, we started to verify the SFC orchestration scenario. We assume that there are five SFCs, which are composed of four different VNFCS in each SFC, including a firewall, load balancing, deep packet inspection (DPI), and NAT. The input data size range of an SFC is from 300 to 500 KB. It is necessary to mention that the comparison result of the proposed scheme and the existing scheme were generated in the same IoT network scenario. The algorithm to be compared in the simulation is natural DQN, which uses a deep neural network without the federated learning process.

**Figure 8:**
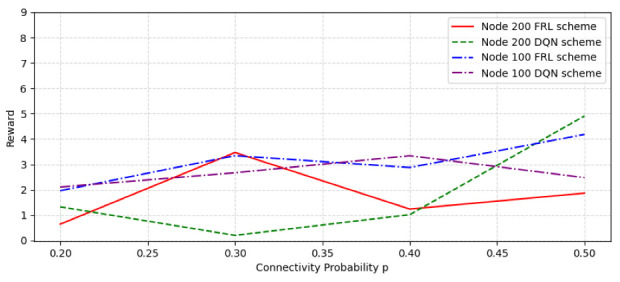
The reward comparison with different node number and connectivity probability.

We test SFC orchestration in random networks constructed with 100 nodes and 200 nodes under different network parameters, respectively. Figure 8 shows the rewards comparison in a random network of different nodes and connectivity probability. The connectivity probability range is from 0.2 to 0.5. As can be seen from the figure, in the case of 100 nodes, the overall reward value of the federal learning algorithm is higher than just using the DQN algorithm; in the case of 200 nodes, when the connectivity possibility is 0.3, the federated learning algorithm reached the highest reward value, significantly higher than the reward value of the DQN algorithm, and after the connection probability is 0.4, the reward value begins to reduce.

**Figure 9:**
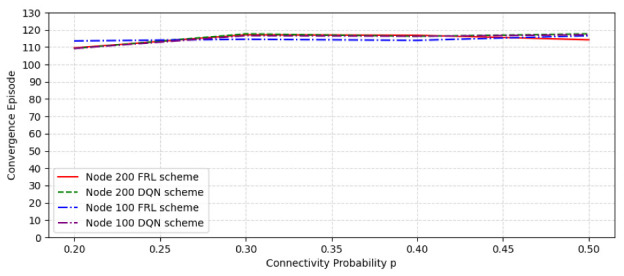
The convergence episode comparison with different node number and connectivity probability.

Figure 9 shows the convergence episode comparison in a random network with different node numbers and connectivity probability. It can be seen that the convergence speed of the federated learning algorithm is faster than that of the DQN algorithm on the whole, whether it is 100 nodes or 200 nodes. With the increase in the number of nodes and connection possibility, the convergence speed of the federated learning algorithm is still faster than the DQN algorithm.

**Figure 10:**
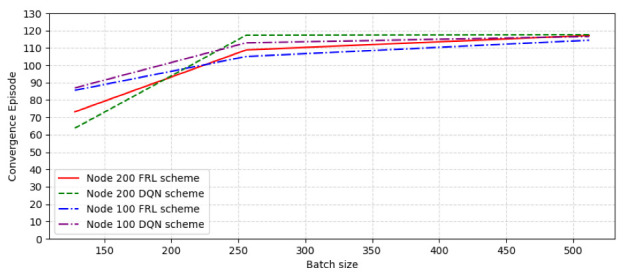
The convergence episode comparison with different node number and batch size.

**Figure 11:**
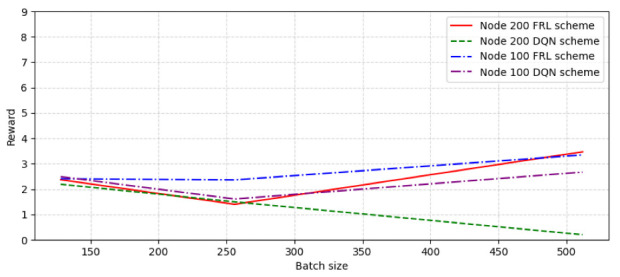
The reward comparison with different node number and batch size.

Considering that different batch sizes may also have an impact on performance, the reward value and convergence speed under different batch size conditions are analyzed. We choose the connection possibility as 0.3 to test the convergence performance when the number of nodes is 100 and 200, and the batch size is 128, 256, and 512, respectively. Figure 10 shows the convergence episode comparison with different node number and batch size. As can be seen from the figure, with the increase in batch size, the convergence rate decreases continuously, but the convergence rate starts to slow down when the size is 256 and tends to balance. Figure 11 shows the reward comparison with different node number and batch size. It can be seen that the reward value of the FRL algorithm increased as the batch size increased, and the reward value of the FRL algorithm compared with the DQN algorithm obviously reached the highest value when the batch size is around 512.

The experimental data show the proposed FRL algorithm can achieve better latency and convergence performance than the DQN algorithm in random network scenarios. When the number of nodes and the connection possibility increases, the overall latency is reduced by about 33%, and the overall convergence speed is improved by about 9%. The federated learning algorithm reduces the communication data amount and communication times between the edge cloud node and the core cloud node, which can effectively reduce the communication overhead, improve the communication efficiency and shorten the delay time [53].

## 5. Conclusions and Future Work

In this paper, a dynamic SFC orchestration security algorithm based on the federated block coordinated descent algorithm is proposed for EC-IoT scenarios. With the goal of minimizing delay, the core cloud server is used to deploy the FRL agent, and the edge node is used to deploy the DRL agent. Combined with block coordinated descent algorithm and the quadratic penalty algorithm, the data privacy protection of the edge computing nodes is realized on the basis of ensuring communication efficiency. This algorithm adopts a random network scenario similar to an IoT scenario to conduct experiments. Compared with the original method of deploying the DRL agent in a core cloud node, this algorithm can achieve better convergence performance in the case of privacy security of edge nodes.

For our next step, we plan to deploy an experimental environment closer to the actual application scenario [54], and the optimization goal will be expanded from meeting the minimum time delay demand to the multi-objective optimization scene, such as minimizing cost and energy consumption, quality of service, and so on. We will also combine the strategy network mechanism with the suitable optimization algorithm to achieve better optimization results, and to improve the generalization of the network model to adapt to the changing EC-IoT application scenarios. In terms of security, we will try to combine the advantages of blockchain and federated learning to achieve a more secure SFC orchestration.

## Figures and Tables

**Table 1:** The parameter setting in the experiment.

Parameter	Value
Numbers of SFC	5
Number of IoT nodes	100/200
Input data size of an SFC	300–500 KB
Computation capacity of IoT node	1–8 GHZ
Memory of IoT node	4–64 GB
Bandwidth between IoT nodes	30–50 Mbps

## Data Availability

Not applicable.

## Abbreviations

IoT	Internet of Things
EC	Edge Computing
NFV	Network Functions Virtualization
VNF	Virtualized Network Function
SFC	Service Function Chain
SDN	Software Defined Network
QoS	Quality of Service
DQN	Deep Q-Network
DDPG	Deep Deterministic Policy Gradient
BCD	Block Coordinated Descending
DRL	Deep Reinforcement Learning
A3C	Asynchronous Advantage Actor-Critic
FRL	Federated Reinforcement Learning
LPWAN	Low-Power Wide-Area Network
